# Classification Framework for Medical Diagnosis of Brain Tumor with an Effective Hybrid Transfer Learning Model

**DOI:** 10.3390/diagnostics12102541

**Published:** 2022-10-20

**Authors:** Nagwan Abdel Samee, Noha F. Mahmoud, Ghada Atteia, Hanaa A. Abdallah, Maali Alabdulhafith, Mehdhar S. A. M. Al-Gaashani, Shahab Ahmad, Mohammed Saleh Ali Muthanna

**Affiliations:** 1 Department of Information Technology, College of Computer and Information Sciences, Princess Nourah bint Abdulrahman University, P.O. Box 84428, Riyadh 11671, Saudi Arabia; 2 Rehabilitation Sciences Department, Health and Rehabilitation Sciences College, Princess Nourah bint Abdulrahman University, P.O. Box 84428, Riyadh 11671, Saudi Arabia; 3 College of Computer Science and Technology, Chongqing University of Posts and Telecommunications, Chongqing 400065, China; 4 School of Economics & Management, Chongqing University of Post and Telecommunication, Chongqing 400065, China; 5 Institute of Computer Technologies and Information Security, Southern Federal University, 347922 Taganrog, Russia

**Keywords:** a brain tumor, hybrid transfer learning, machine learning, deep learning, magnetic resonance imaging

## Abstract

Brain tumors (BTs) are deadly diseases that can strike people of every age, all over the world. Every year, thousands of people die of brain tumors. Brain-related diagnoses require caution, and even the smallest error in diagnosis can have negative repercussions. Medical errors in brain tumor diagnosis are common and frequently result in higher patient mortality rates. Magnetic resonance imaging (MRI) is widely used for tumor evaluation and detection. However, MRI generates large amounts of data, making manual segmentation difficult and laborious work, limiting the use of accurate measurements in clinical practice. As a result, automated and dependable segmentation methods are required. Automatic segmentation and early detection of brain tumors are difficult tasks in computer vision due to their high spatial and structural variability. Therefore, early diagnosis or detection and treatment are critical. Various traditional Machine learning (ML) techniques have been used to detect various types of brain tumors. The main issue with these models is that the features were manually extracted. To address the aforementioned insightful issues, this paper presents a hybrid deep transfer learning (GN-AlexNet) model of BT tri-classification (pituitary, meningioma, and glioma). The proposed model combines GoogleNet architecture with the AlexNet model by removing the five layers of GoogleNet and adding ten layers of the AlexNet model, which extracts features and classifies them automatically. On the same CE-MRI dataset, the proposed model was compared to transfer learning techniques (VGG-16, AlexNet, SqeezNet, ResNet, and MobileNet-V2) and ML/DL. The proposed model outperformed the current methods in terms of accuracy and sensitivity (accuracy of 99.51% and sensitivity of 98.90%).

## 1. Introduction

The brain and spinal cord are two main primary control centers of the human body; hence, any harm to this region is considered extremely dangerous. Tumors are the formations of abnormal tissue that can occur in any part of the human body [1]. One of the distinguishing features of brain cancer is abnormal development of cells of the brain or spinal cord. Because of their different natures, malignant and benign brain tumors require different treatments. Based on benignity and malignancy, the World Health Organization (WHO) has classified brain cancers into four major groups (Grade I–IV). Malignant brain tumor of grades III and IV grow quickly, metastasize (body-wide spread), and have a negative impact on healthy cells. Furthermore, the rigidity of the skull and the additional development of brain cells may result in pressure within the skull, which can harm the brain. Surgery, chemotherapy, radiation, and various treatment combinations are among the most advanced therapies available today. Even with the most intensive medical supervision, patients frequently do not live for more than 14 months [2]. As a result, early brain tumor detection is a crucial step in meticulously planning therapy. Patients’ chances of survival may improve if the BT diagnosis early. Computer tomography (CT) and magnetic resonance imaging (MRI) are two methods commonly used in the diagnosis and evaluation of brain tumors. MRIs use magnetic fields rather than X-rays, as in CT scans, to obtain a comprehensive image of the body. These techniques provide medical professionals with an in-depth view of information about the inside of the body, assisting them in determining the presence and location of symptoms. As a result, early BT detection and classification allows medical professionals to better plan appropriate therapy using MRIs as well as other imaging modalities [3]. Furthermore, pituitary tumors, gliomas, and meningiomas represent the primary subtypes of BT. Pituitary tumors are usually harmless and develop in the pituitary glands, which are located in the brain’s basal layer and are responsible for the production of many important hormones [4]. A glioma is a malignancy that develops when glial cells grow uncontrollably. Typically, such cells work in nerves and facilitate the function of the central nervous system. Gliomas typically develop in the brain, but they can also develop in the spinal cord [5]. Cancers known as meningiomas can develop on the membrane that serves as a protective covering for the brain and spinal cord [6]. Identification of BT requires the ability to differentiate between normal and abnormal brain tissue. Differences in shape, position, and size increase the difficulty of detecting BT, but it remains a challenge that must be overcome. BT analysis makes use of some of the most fundamental aspects of medical image processing, including classification, segmentation, and detection [7]. During the preliminary stages of treatment, the BT classification is a crucial step in identifying the specific kind of tumor that is currently present. The field of biomedical image processing has several cutting-edge computer-aided diagnostic tools to help radiologists with patient guidance and improve BT classification accuracy [8]. When high-grade tumors are present, brain tumors are a dangerous condition that significantly reduces a patient’s life expectancy. To be more specific, BT diagnosis is critical for treatments which have not improved the patient’s quality of life [9]. The proposed CAD system is intended to work under the supervision of a radiologist [10], and accurate tumor segmentation is required for better cancer identification.

In addition, the diagnosis of brain tumors in clinics relies on the visual inspection of patients by pathologists, who are highly trained experts in the field of neurosurgery. However, this procedure is done manually, which is not only time-consuming but also tedious and highly susceptible to pathologist error. This is due to the fact that the majority of cells typically comprise a portion of random, arbitrary, and uncontrolled visual angles.

It is essential to identify the type of tumor that a patient has, including whether it is glioma, pituitary, or meningioma. The purpose of this study is to differentiate between the three categories. Early diagnosis of the three types of BTs is essentially based on the neurosurgical point of view.

To address the information issues above, various ML/DL models for BT segmentation have been performed specially, deep transfer learning model (DTL) [11,12,13,14]. In DTL, the GoogleNet model has recently gained a lot of popularity in the field of medical imaging classification and grading. It is widely utilized for interacting with raw images and performing a better classification. Moreover, a transfer learning model such as AlexNet layers are used extensively in the process of extracting its features [15]. AlexNet is the most used deep transfer learning (TL) model, and the primary application it serves is image categorization [16]. Due to the different advantages of both models, the researchers were motivated to use the hybrid deep transfer model to improve the accuracy and performance of current algorithms in identifying different types of brain tumors, as well as to evaluate the approach using a dataset that was freely available to the public. Furthermore, the research question of this study is “how accurately and effectively can the hybrid TL system recognize and categorize various types of BT diseases?”. The following is a list of our key contributions to this research:

- A precise Computer Aided Diagnosis system for BT is presented using deep learning.
- A new hybrid deep learning approach, GN-AlexNet, is introduced for the classification of three types of brain tumors (pituitary, meningioma, and glioma). The proposed CAD system is thoroughly tested on a publicly available benchmark dataset of Contrast-Enhanced magnetic resonance images (CE-MRI).
- In terms of accuracy and sensitivity, the proposed model performed significantly better than the existing techniques (with an accuracy of 99.51% and a sensitivity of 98.90%).
- High classification performance has been achieved with the suggested model, together with decreased time complexity (ms). The GA-AlexNet classifier is used for successful BTs diagnosis in clinical and biomedical research.

The paper has the following outline. Section 2 discusses the literature review. Model specifications are presented in Section 3. Discussion and results are presented in Section 4. The final section presents the study’s findings and suggestions for future study.

## 2. Literature Review

Many machine learning and deep learning models [17,18,19,20,21,22,23,24,25] were used to classify and detect anomalies in biological images.The model presented in [26] outlines a process for the early diagnosis of brain tumors that involves the extraction and concatenation of many levels of features. Inception-v3 and DensNet201 are the three deep learning models that were used to verify this model before it was trained. These two models were used to investigate two distinct potential courses of action for the classification of BT. Using a dataset consisting of 253 images and pre-trained versions of VGG-16 and Inception-V3, a model for the automated identification of brain tumors was developed [26] and presented. This dataset includes 155 pictures of cancerous tumors and 98 images of healthy tissue. It was unable to fine-tune the CNNs using the dataset since it was not large enough, and the test dataset was also too small to check the performance of the model.

Using VGG-16 and the BRaTs dataset, a model for the automated identification of brain tumors was suggested [27]. Through the utilization of transfer learning and fine-tuning carried out across a total of 50 epochs, the accuracy of the model was brought up to 84%. Srivastava et al. presented a dropout strategy as a solution to the problem of overfitting in neural networks [28]. This technique involves randomly removing units and the connections between them.

P. Dvorak et al. in [29] selected a convolutional neural network as the learning method because it is effective at handling feature correlations using the freely available BRATS2014 data set, which contains three distinct multimodal segmentation tasks They evaluated the method, and they were able to obtain state-of-the-art results for the brain tumor segmentation data set, which included 254 multimodal volumes and required only 13 s per volume to process. Artificial Convolutional Neural Networks were implemented by Irsheidat et al. in their research paper [30]. The results indicated that the CNN model obtained satisfactory results.

In order to analyze MRI images, this model is capable of performing convolutional operations on the raw data. This neural network accurately predicts the presence of brain tumors because it was trained with MRI scans of 155 healthy brains and 98 tumors. Sravya et al. [31] researched the identification of brain tumors and provided several significant issues and methodologies. Using the YOLO model and the deep learning package FastAi with the BRATS 2018 dataset, which included 1992 MRI images of the brain, an automated brain tumor identification method was developed and investigated [32]. The accuracy of YOLO was measured at 85.95%, whereas the accuracy of the FastAi classification model was measured at 95.78%.

The VGG-16 model used for brain tumor detection was proposed [33] to identify MRI pictures as either tumorous or non-tumorous. The training was done using the Kaggle dataset, and the authors demonstrated an increase in accuracy as a result. Nevertheless, the authors trained the model in its entirety.

M.O. Khairandish et al. [34] proposed hybrid model combined CNN and support vector machine (SVM) for brain MRI image classification. The proposed study conducted comparative studies of segmentation with different DL model. The proposed approach performed best classification up to 99%.

For BT detection and classification, Swati et al. [35] implemented a CNN based block-wise-tuned (BFT) system. In comparison to the manually constructed features, the fine-tuned VGG-19 using the BPT approach obtained 94.84% classification accuracy in a shorter amount of training time. Kumar et al. [36] presented the Dolphin-SCA algorithm, which is a novel optimized DL method, for the detection and categorization of BT. Dolphin-SCA is an example of a novel optimized DL method. The mechanism makes use of a deep convolutional neural network. For the purpose of segmentation, the researcher employed a fuzzy deformable fusion model in conjunction with a sine cosine algorithm that was based on dolphin echolocation (DolphinSCA). A deep neural network that used Dolphin-SCA as its foundation and was based on power statistical features and LDP was used to make use of the retrieved features. The deep neural network was constructed using LDP. When using the method that was suggested, the accuracy of the categorization was found to be 96.3%. Deepak et al. [37] employed a pre-trained version of GoogleNet for feature extraction, and they relied on tried-and-true classifier models for both BT classifications. In comparison to the most recent and cutting-edge methodologies, the suggested technique achieved an accuracy of 98%. A hybrid model for the classification of BT was presented by Raja et al. [38], which takes into account a number of different backgrounds (namely, pre-processing by using a non-local express filter and segmentation by using the Bayesian fuzzy technique). After that, the scattering transform, the wavelet packet Tsallis entropy approach, and the theoretical measures were used to extract several aspects of the picture that had previously been measured. An accuracy of 98.5% was achieved in the classification process by utilizing a hybrid strategy that was found on a combination of a softmax and a deep autoencoder. Ramamurthy et al. [39] developed a novel method for the detection of BT that is based on DL. They have optimized the process using the Whale Harris Hawks framework. Following the segmentation of the tumors in the images using cellular automata, several parameters including the mean, size, kurtosis, and variance are retrieved. Following this, the components are classed for improved brain tumors identification using an optimization method that was provided by Whale Harris Hawks. Using a skull skipping algorithm, in conjunction with a support vector machine (SVM) classifier, the approach that was suggested achieved an accuracy rate of 81.6%, which was the highest it had ever achieved. The Berkeley wavelet transformation (BWT) and segmented characteristics were used by Bahadure et al. [40] which successfully identify brain tumors (BT) (contrast, texture, shape, and color). The skull skipping algorithm was utilized to accomplish this goal, which resulted in the removal of non-brain parts from MR images. The results of the experiment showed that there was a 96.51% chance that they were accurate. Waghmare et al. [41] used of several distinct CNN architectures in order to recognize and classify brain tumors. The classification accuracy of the expanded data set was improved by fine-tuning the VGG-16 algorithm, and it achieved the highest level of accuracy that was appropriate, which was 95.71%. This was the highest level of classification accuracy that could be achieved.

The current state of the art in using deep learning to classify MRI images of brain tumors shows the network’s higher performance in correctly and precisely classifying brain tissues, indicating its wider usage in this field and how transfer learning contributes to high accuracy in brain MRI segmentation. From the above literature its clearly shows that the TL model performs better than other DL models in brain tumor detection and classification. In order to further improve the classification performance of BTs, we used a hybrid transfer learning model of AlexNet and GoogleNet to create a hybrid deep learning model for the segmentation and classification of three types of brain tumors (pituitary, meningioma, and glioma).

## 3. Methodology

**Figure 1:**
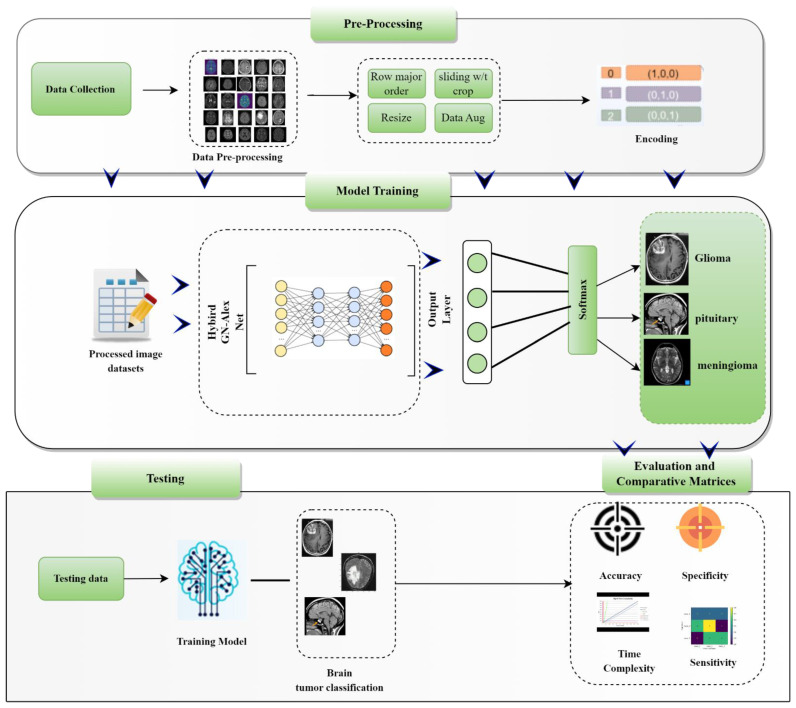
The block diagram of the framework for BT classification using the proposed GN-AlexNet deep learning model.

This section describes the methodology and the dataset, including the CE-MRI dataset and its pre-processing, as well as the proposed GN-AlexNet deep learning model for classifying CE-MRI data set images into BT tumor tri-classification. The main modules in the framework of developing and testing the proposed CAD system for BT classification are shows in Figure 1. Furthermore, the performance indicators (Accuracy, Precision, Sensitivity, Specificity) display the classification performance of the proposed model.

### 3.1. Brain Tumor CE-MRI Dataset

**Figure 2:**
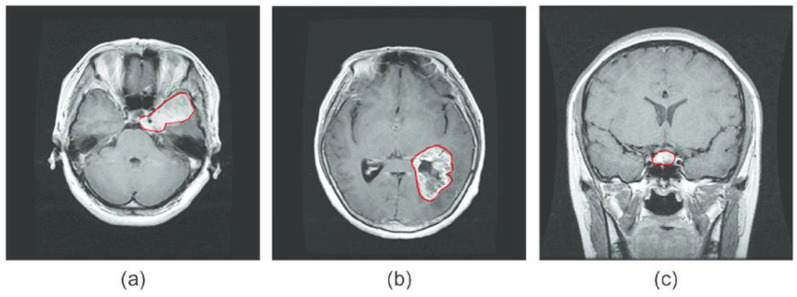
MRI images of three categories (**a**–**c**) (glioma, pituitary, and meningioma).

To conduct their research, the authors used a publicly available MRI dataset. [42]. The TJU Hospital in China gathered brain MRI scans from 262 patients. There are 3062 brain MRI images in total, including 1426 gliomas, 760 meningiomas, and 940 pituitary tumor images. Figure 2 shows examples of the various classes of BT images. The image is a 2D volume with 512 × 512 rs and a size of 0.49 × 0.49 mm^2^. The dataset format is available online in .mat in figshare. In this study, 2146 MRI images (70%) were used for training, while 918 were used for testing (30%). Table 1 contains detailed information about the CE-MRI data set, such as the number of images, patients, and class label for each type of brain cancer (glioma, pituitary, and meningioma) as shown in Figure 2.

### 3.2. Data Preprocessing and Augmentation

This dataset includes 3075 images of different types of BTs. Each image was transformed to a grayscale format. These data, which have been preprocessed, are one of the things that the neural network uses as input, along with the label of the image. Label 1 describes an image of a glioma; Label 2 depicts the pituitary gland, and Label 3 depicts a meningioma. However, a dataset consisting of 3075 MRI images is needed, in order to train the hybrid deep transfer learning (GN-AlexNet) model effectively, which has one million parameters. Data augmentation is the approach that needs to be taken to solve this issue. By rotating, scaling, and adding noise to the data that already exists, this method can be used to artificially increase the size of the data. The data can be magnified by zooming in on the image, rotating it horizontally or vertically by a predetermined angle, and adjusting the brightness range upwards or downwards, respectively. The MRI images have been augmented by utilizing all these methods. Because of the application of the augmentation methods, the data size was increased by a factor of 16, and as a result, the issue of overfitting was resolved [11].

### 3.3. Proposed Model

Within the scope of this study, we propose a hybrid transfer learning model that combines AlexNet and GoogleNet to produce a hybrid deep learning model for the segmentation and classification of three distinct types of brain tumors (pituitary, meningioma, and glioma). First, in this section, we go over AlexNet and GoogleNet, and then, the specifics of the hybrid GN-AlexNet deep learning model are described.

#### 3.3.1. AlexNet

**Figure 3:**
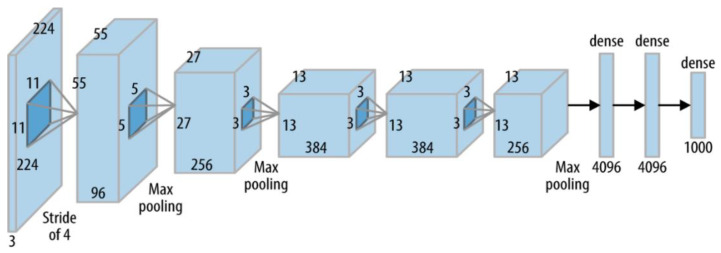
The basic block diagram of AlexNet model.

The AlexNet model was developed by Alex Krizhevsky et al. [43]. On 30 September 2012, AlexNet participated in ImageNet Large-Scale Visual Recognition Competition. The top-5 error rate for the network was 15.3%, which was more than 10.8 points lower than the runner-up. The primary finding of the original study was that the model’s depth was crucial for its greater efficiency, which was computationally expensive but made achievable by the use of graphics processing units (GPUs) in training, as seen in Figure 3.

In addition to this, elevating the total number of convolution layers in the AlexNet resulted in the production of features that were more specific, precise, and robust. In contrast to the first layer, which only recovered low-level features, the subsequent two convolutional layers were able to extract high-level characteristics. The Max-pooling layer helped to improve the accuracy towards the end of the network as shown in Figure 3.

#### 3.3.2. GoogleNeT

The block of the GoogleNet and their fundamental layers are what makes up the hybrid deep (GN-AlexNet) model. The AlexNet layer is also a part of the model. Training a convolution network is a challenging task in the beginning, and the procedure can sometimes take as much as a few hours. Therefore, rather than beginning the process of training a new deep learning classifier, it would be preferable to train the proposed model using a classifier.

This would be the preferred method. Considering GoogleNet’s [16] success in the ILSVRC (2014) ImageNet competition, we decided to make it the cornerstone of our own research. In total, GoogleNet consists of 144 layers, with only 22 of those layers being learnable. Two convolution layers, four maximum pooling layers, one average pooling layer, two normalization layers, one completely connected layer, and nine inception layer modules are included in these layers. Additionally, each inception module contained one MXs layer in addition to the six CLS that were standard. The GoogleNet algorithm has been updated with a new input layer with dimensions of 224 × 224 × 1. The ReLU activation function was utilized within the framework of the pre-trained GoogLeNet methodology. Throughout the procedure, the ReLU activation function disregarded any values that were in the negative range and replaced them with zero. Leaky ReLU, an improved version of ReLU that replaces all negative values with positive ones, is another option [44]. During the process, the Deep Transfer learning model classifier that had initially been developed lost the last five layers of the GoogleNet classifier that it had been using. After they were removed, ten new layers were added in their place. In addition to this, the Leaky ReLU activation function was applied to the ReLU activation function that was present in the feature map layer to enhance the expressiveness of the model that was proposed and to discover a solution to the dying ReLU problem.

**Figure 4:**
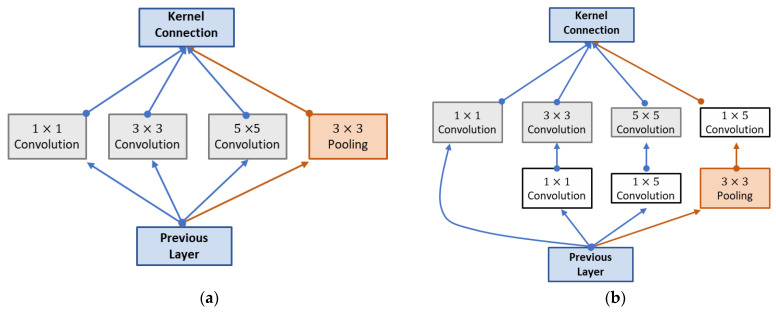
GoogleNet’s inception module. (**a**) Inception module with no convolution layer. (**b**) A 1 × 1 convolution layer in inception “Reprinted with permission from Ref. [15]. 2022, Samee et al.”.

Three methods from the NIN (Network In Network)—inception modules, global average pooling, and the 11 Convolution—have been implemented in GoogleNet. Figure 4a depicts an inception module, which consists of a max pooling layer of size 33 and three convolutional layers of size 11, 33, and 55 that operate in tandem. The inception module accepts data from a lower layer, processes it using parallel operations, and sends the combined resultant feature maps on to the next layer. Using this method, you can expand your network. As seen in Figure 4b, an 11 convolution applied to the inception module’s internal layers significantly reduced the module’s processing requirements.

It was possible to do this without changing the fundamental structure of the convolution neural network. After these modifications were made, the total number of layers increased to 154, from 144 previously. A filter (patch) size of 8 × 8 was used in the first convolution layer, which greatly reduced the image size very soon. The 1 × 1 convolution block was used in the convolutional network’s second layer, which had a depth of 2. Dimensionality reduction was the goal, so this was done. In addition, GoogleNet’s inception module makes use of a number of convolution kernels, including 1 × 1, 3 × 3, and 5 × 5, to extract features at a range of granularities, from the tiniest details to the most fundamental aspects [45].

The greater the convolution kernel, the more surface area it covers while computing the features. Similarly, the 1 × 1 convolution kernel provides more information while also reducing the amount of processing required. Four convolutional layers with a very small filter size of 1 × 1 have been included as part of the recent updates as shown in Table 2.

#### 3.3.3. The Hybrid GN-AlexNet Deep Learning Model

**Figure 5:**
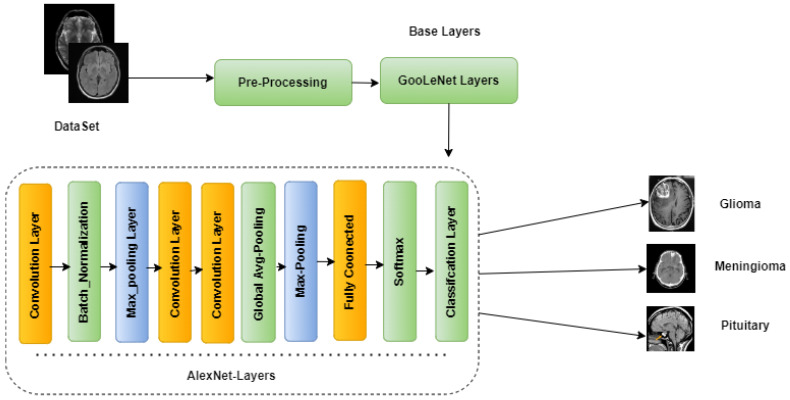
The proposed GN-AlexNet.

In addition, the ReLU activation function in the feature map layer was modified to improve the expressiveness of the proposed model and get over the dying ReLU issue. As a consequence of this, the proposed model was in a role to extract more comprehensive, discriminative, and deep features than the state-of-the-art pre-trained deep learning models that were mentioned earlier. This resulted in improved classification performance, which can be seen in Figure 5.

The proposed hybrid learning model (GN-AlexNet), has different layers including: the input, convolutional, activation function, normalization, Max_Pooling, fully connected, softmax, and classification layers. The input layer receives the images as input. In the GN-AlexNet learning model, the input images have a size of 224 × 224 × 1. Those three digits represent the image’s width, height, and channel size in grayscale (1 for grayscale images and 3 for color images). The images were first sent to an input layer before any further processing could begin. The convolutional layer involves a mathematical operation that requires two inputs: the input image matrix and a filter. The input image was multiplied by the filter, and a feature map was generated as an output. The mathematical expression of the convolution layer is as follows in Equation (1).

(1) $$Z_{b}^{a}=\sum_{i\in dc}k_{aj}^{k}*y_{l}^{c-1}+a_{d}^{c}$$

where *K* represents the number of layers, $a_{d}^{c}$ shows bias, and the feature map is both layers represented by $Z_{b}^{a}$ to *c* − 1 of the layer *d*.

The activation layer includes an activation function that gives nonlinearity to the neural network. Rectifier linear units (ReLUs) are used because they increase the training speed. Equation (2) shows the mathematical equation for the ReLU activation.

(2) $$R\left(x\right)=\{\begin{array}{c} x\;\;\;\;\;\;\;\;\;\;\;\;\;\;\;\;\;\;\;\;if\;x>0\\ 0\;\;\;\;\;\;\;\;\;\;\;\;\;\;\;\;\;\;\;\;if\;x\leq 0 \end{array}$$

To normalize the parameters generated by the proposed convolution layers, a batch normalization layer is applied to the outputs. The proposed model training period is shortened as a result of normalization, which makes the learning process both more effective and more rapidly accomplished.

The main limitation of the convolutional layer is that it captures features that depend on their location. Thus, if the location of the feature in the image changes slightly, the classification becomes inaccurate. Max-Pooling allows the network to overcome this limitation by making the representation more compact so that it is invariant to minor changes and insignificant details. Max pooling and average pooling were used to connect the features.

The fully connected layer receives the features learned in the convolutional layers. When a layer is said to be “fully connected”, it means that all of its nodes are linked to those in the next layer. This layer’s key focus is to label input images with their respective classes. A softmax activation function is used in this layer.

The Loss function (H) must be minimized during training. The output is calculated after the image has passed through all the previous layers. It is compared to the desired output using the loss function, and the error rate is calculated. This process is repeated for several iterations until the loss function is minimized. The loss function were used categorical cross-entropy (CCE). Equation (3) shows the mathematical equation for CCE.

(3) $$H=-H=-\sum_{m=1}^{M}y_{m}^{l}\cdot \;k\log{\hat{y}}_{m}^{j}$$

where ${\hat{y}}_{m}^{j}$ represents predicted label and $y_{m}^{l}$ represents target label of sample *m* among *M* number of samples.

The activation function causes further normalization of the fully connected layer’s output. The probabilistic computation is carried out by the network, and softmax generates the output in positive values for each category. The classification layer is the final layer of the model to be shown. This layer is responsible for generating the output by combining all of the inputs. A probability distribution was obtained as a consequence of the use of the softmax activation function. Table 2 lists the layers used in the proposed model, as well as their specifications.

### 3.4. Experimental Setup

This research implemented a wide range of extensive libraries while conducting experiments, including Tensorflow, Pandas, Numpy, and Keras. The proposed model is being trained on Keras while also running Python 3.6. To validate the performance of our proposed framework, analytical simulations are run on a computer equipped with a cori7-processor and a Graphical Processing Unit (GPU). These simulations are carried out using an Intel CPU.

### 3.5. Performance Evaluation Metrics

To assess the performance of brain tumor detection, evaluation metrics must be performed, including all of the framework’s available parameters. Although there is no standardized measure that can be used to classify performance matrices, the key performance matrices Accuracy (Acc), Sensitivity (Sens), Precision (Prec), and Specificity (Spec) are used relatively frequently.

This performance matrix was used to investigate the feasibility of the proposed model. The accuracy of a model is entirely dependent on several critical metrics, including the True Positive Rate (TPR) and the True Negative Rate (TNR), as well as the False Positive Rate (FPR) and the False Negative Rate (FNR). Equations (4)–(7) describe the key performance indicators that have been employed in this study.

(4) $$\mathrm{Acc}=\frac{\mathrm{TP}+\mathrm{TN}}{\mathrm{TP}+\mathrm{TN}+\mathrm{FP}+\mathrm{FN}}$$

(5) $$Prec=\frac{\mathrm{TP}+\mathrm{TN}}{\mathrm{TP}+\mathrm{FP}}$$

(6) $$Sens=\frac{\mathrm{TP}+\mathrm{TN}}{\mathrm{TP}+\mathrm{FN}}$$

(7) $$Spec=\frac{2*\mathrm{TP}}{2*\mathrm{TP}+\mathrm{FP}+\mathrm{FN}}$$

## 4. Result and Discussion

**Figure 6:**
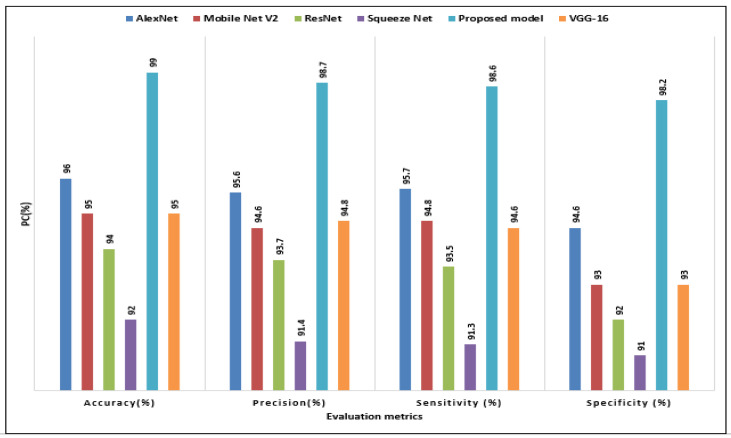
The performance evaluation of the proposed GN-AlexNet model versus the transfer learning (pretrained models).

This section provides an evaluation of the GN-AlexNet models performance in comparison to other transfer learning models, including VVG-16, AlexNet, SqeezNet, ResNet, and Mobile Net V2, utilizing key performance indicators for the purpose of detecting and classifying brain tumors. Accuracy is an important component that demonstrates the specific class efficiency. Furthermore, the precision represents the ratio of accuracy in real-time tumor class prediction. While specificity is used to detect non-tumor classes. The Acc, Pres, Sens, and Spec of the proposed model and other transfer learning methods were compared in this study. Figure 6 compares the GN-AlexNet model’s attained Acc, Pres, Sens, and Spec to the other transfer learning models. As shown in the bar chart, the classification performance of the proposed model revealed that it outperformed other TL models in terms of Acc (99.10), Pres (99%), Sens (98.90%), and Spec (98.50%). As shown in the Figure 5, the SqueezNet model has the lowest performance measure.

**Figure 7:**
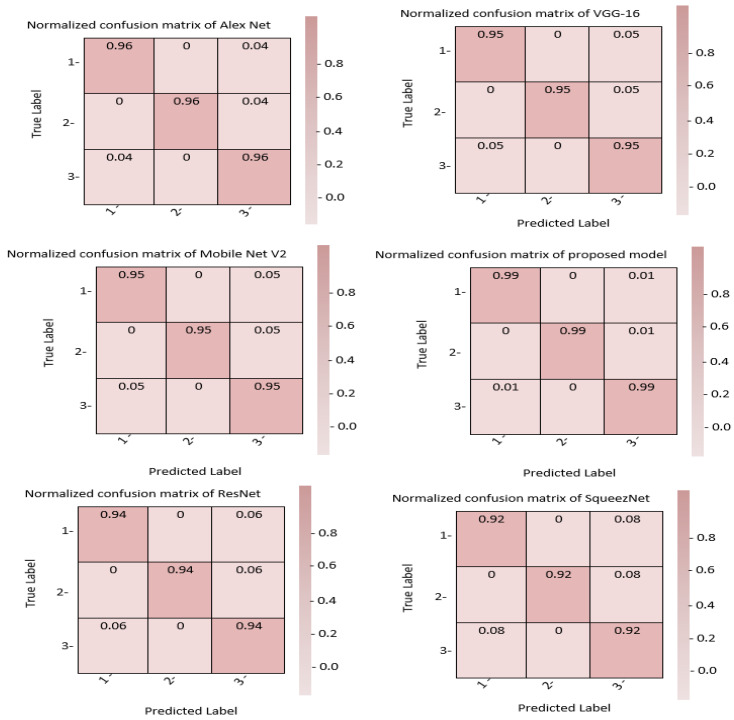
Presents the confusion matrix of the proposed transfer learning techniques in brain tumor detection.

When assessing how well an evaluation indicator performs, a confusion matrix can be used to quantify how well each class is classified. The proposed GN-AlexNet showed excellent tri-tumor type detection and accurate classification of each brain tumor type in this experiment, as measured by the accuracy of their confusion matrices. Figure 7 displays that the transfer learning model has the lowest performance measure.

**Figure 8:**
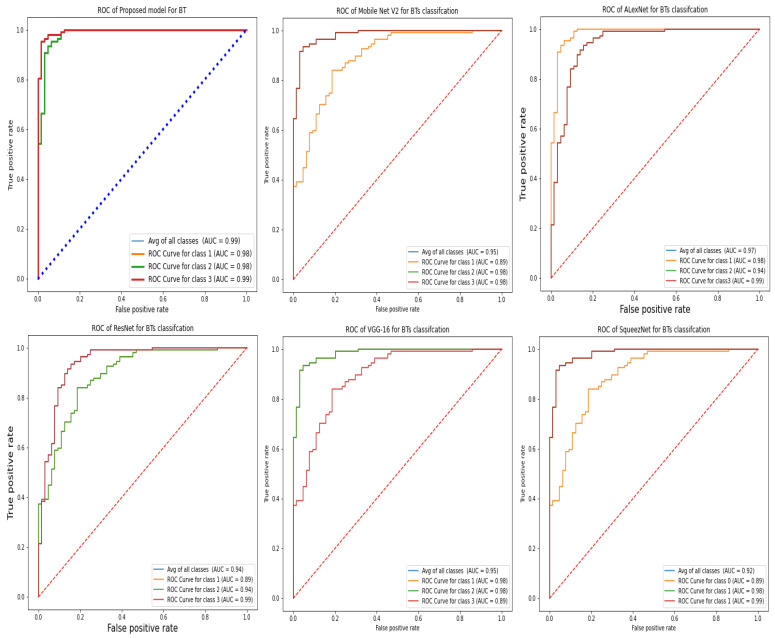
The ROC of the proposed model with transfer learning techniques in brain tumor detection.

The ROC curve is an essential tool for assessing whether a system is successful in the detection of brain tumors. Based on the ratio of the true positive rate (TPR) to the false positive rate (FPR), the ROC curve illustrates how well each classification can detect a given variable (TPR). Figure 8 shows the receiver operating characteristic (ROC) curve for the proposed model performs exceptionally well in comparison to other transfer learning methods.

**Figure 9:**
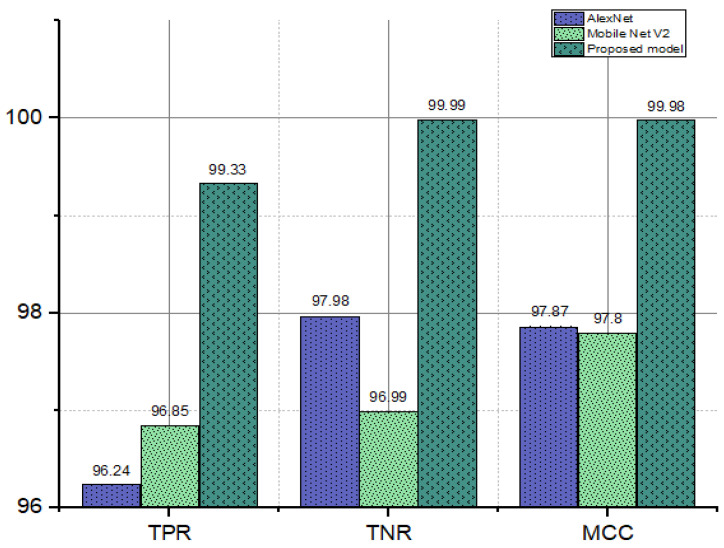
Bar chart of the attained values of the TPR, TNR, and MCC of the proposed model and the other TL models.

To improve the proposed model even further, we compared it to the top-performing TL model using the confusion matrix’s key performance indicators: the True Positive Rate (TPR), True Negative Rate (TNR), and Matthews Correlation Coefficient (MCC) (Alexnet and MobileNet-V2). As can be seen in Figure 9, the TPR, TNR, and MCC values are all highest for the proposed model.

### 4.1. FDR, FNR, FOR, and FPR Analysis

In addition, the proposed hybrid DTL model outperforms the state-of-the-art transfer learning models on the current dataset across a wide range of performance metrics. These metrics include the false positive rate (FPR), the false negative rate (FNR), the false omission rate (FOR), and the false detection rate (FDR) are as follows:

(8) $$FDR=\frac{\mathrm{FP}}{\mathrm{FP}+\mathrm{TP}}$$

(9) $$FPR=\frac{\mathrm{FP}}{\mathrm{FP}+\mathrm{TN}}$$

(10) $$FNR=\frac{\mathrm{FN}}{\mathrm{FN}+\mathrm{FP}}$$

(11) $$FOR=1-\frac{\mathrm{FN}}{\mathrm{TN}+\mathrm{FN}}$$

FDR = the proportion of patients who have a positive test result, despite the fact that the fundamental condition is negative, is a false discovery rate.

FPR = the number of individuals who have a condition that is known to be negative but for which the test nonetheless returns a positive result is known as the false positive rate.

FNR = the false negative rate can be defined as the proportion of individuals who have a known positive condition but have a negative test result for that disease.

FOR = The false omission rate can be defined as the proportion of individuals who have a negative test result despite the fact that they actually have a positive condition.

**Figure 10:**
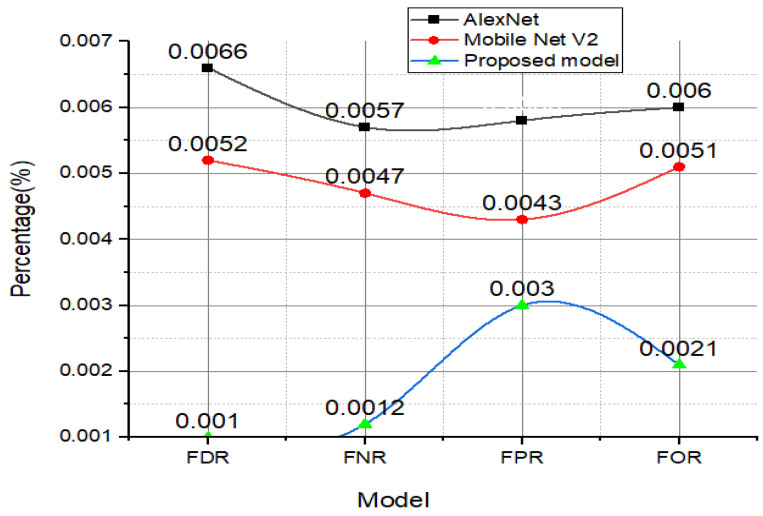
The attained values of the FDR, FNR, FPR, and FOR of the proposed model and the other TL models.

Figure 10 demonstrates that the proposed model possesses impressive performance metrics such as an FPR value of 0.0030%, FOR value of 0.0050%, FDR value of 0.00525%, and FNR value of 0.0012%.

**Figure 11:**
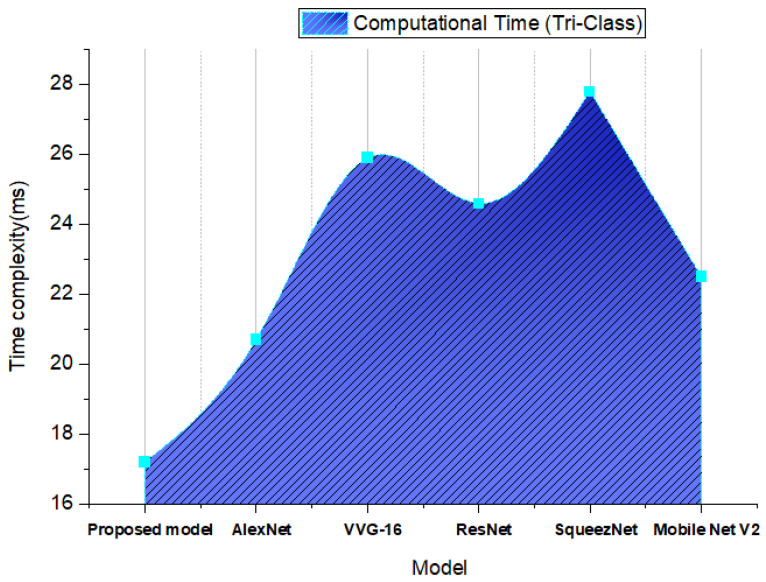
The time complexity of the proposed transfer learning techniques in brain tumor detection.

The training time of a system is an important metric for assessing its performance because it measures how long it takes for a system to absorb the sustainability of its relative features. In this study, the proposed model’s training time was 16 ms, which is very short when compared to other transfer learning methods, as shown in Figure 11.

### 4.2. Comparative Results with Existing Benchmark

**Figure 12:**
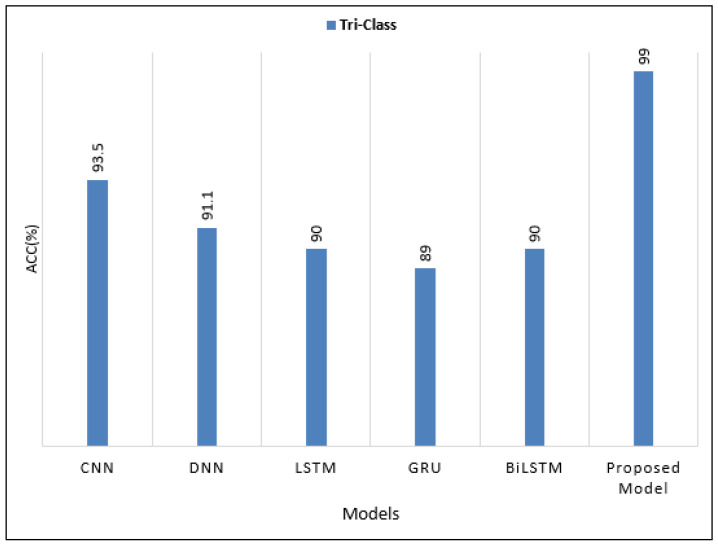
Comparing the attained results of the proposed CAD system for BT classification with state-of-art DL techniques.

The GA-AlexNet that has been proposed has been evaluated alongside other outstanding benchmark algorithms such as LSTM, CNN, GRU, and many others. Its performance was evaluated using Acc, Pres, Sens, and Spec, as shown in Figure 12, so that its performance can be validated.

One of the more well-known applications of machine learning in the medical field is the classification of brain tumors. In order to create a reliable CAD system for these kinds of uses, many researchers and developers have looked into the problem and attempted to create one, with their findings published as a body of work. As shown in Table 3, the proposed system’s classification performance is compared to that of several state-of-the-art breast cancer detection systems. The purpose of this comparison is to gauge how well the modified system.

## 5. Conclusions

This research was conducted with the intention of classifying BTs tri-classification (pituitary, meningioma, and glioma), which was implemented across multiple layers of GoogleNet and AlexNet. The architecture of GoogleNet served as the basis for the development of the GN-AlexNet framework that was proposed. After removing the five layers of GoogleNet, 10 additional layers of AlexNet were added, which extract features and classify different types of BTs automatically. In addition, the ReLU activation function was modified to be a leaky ReLU activation function, but the core architecture of AlexNet was not changed.

On the same CE-MRI dataset, the proposed model was compared to transfer learning techniques (VGG-16, AlexNet, SqeezNet, ResNet, and MobileNet-V2) and ML/DL. The proposed hybrid TL model outperformed the current methods in terms of accuracy and sensitivity (accuracy of 99.51% and sensitivity of 98.90%). ROC (%), time complexity (%), and an extensive metrics approach (FNR, FPR, and MCC values) were used to compare the proposed techniques with previous TL methods and the latest ML/DL model. The proposed hybrid model diagnostic improves each BT class with improved classification performance and less detection time (%). In addition, a future study will show how the hybrid method performs with various data types, such as spotting signs of lung cancer, COVID-19 infection, and pneumonia. Furthermore, the proposed model also needs to be tested on big data.

## Figures and Tables

**Table 1:** A description of the brain data set.

Tumor Class	Images	#Patients	Class Labels	MRI-Images	Testing Data	Training Data
Glioma	1427	90	1	AX(494),CO(437),SA(496)	999	428
Pituitary	940	63	2	AX(291),CO(319),SA(320)	652	278
Meningioma	708	83	3	AX(209),CO(268),SA(231)	495	213
Total	3075	36			2146	918

Axial = AX, Coronal = CO, and Sagittal = SA.

**Table 2:** The specifications of the layers in the proposed learning model, GN-AlexNet.

Layer	Filter Size	No of Filter	Epsilon
Convolution Layer	1 × 1	940	0.002
Batch_norm_layer	-	-	0.001
Soft-Max Layer			
Clip_ReLU_layer			
Group_Conv_layer	3 × 3	940	
Clip_ReLU_layer			0.002
Convolution Layer	1 × 1	300	
Convolution Layer	1 × 1	1260	
Batch_norm_layer			0.002
Glob_AVG_P_layer			
FC layer			
SoftMax			
Classification Layer			

**Table 3:** Comparative study of the proposed model with recent state-of-art methods.

References	Methods	Acc (%)	Prec (%)	Sens (%)	Spec (%)
This work	Proposed model	99.1	99	98.9	98
[46]	CNN	91.6	90.8	89.9	89.5
[47]	BWT+SVM	95.9	94.6	93.8	93.6
[48]	SVM, KNN	96.8	95.2	95	94.6
[11]	AlexNet	94.6	93.6	93	92.4
[47]	GA-CNN	93.9	92.5	92	91.9
[31]	M-SVM	96.8	96	95.8	95
[29]	ANN	94.7	93.5	93	92.50

## Data Availability

The data used to support the findings of this study are available at https://www.mathworks.com/products/matlab.html (accessed on 10 September 2022).
